# GLP-1 Receptor Agonists in Obstructive Sleep Apnea: A Translational Perspective on Mechanisms and Clinical Implications

**DOI:** 10.3390/ijms27167108

**Published:** 2026-08-08

**Authors:** Isabella Gómez-Maldonado, Andrea Rodríguez-Arana, Alejandro Tamayo-Pinzón, Juan Esteban Albornoz-Suárez, María José López-Franco, Juan Andrés Mejia-Manrique, Juan Jacobo Forero-Caycedo, Luis Carlos Rojas-Rodríguez, Natalia Buitrago-Ricaurte, Mariana Gaviria-Carrillo, Jesús Rodríguez-Quintana, Carlos Alberto Calderón-Ospina

**Affiliations:** 1 Research Group in Applied Biomedical Sciences (UR Biomed), School of Medicine and Health Sciences, Universidad del Rosario, Bogota 111221, Colombia; 2 Pharmacology Unit, Department of Biomedical Sciences, School of Medicine and Health Sciences, Universidad del Rosario, Bogota 111221, Colombia; 3 Neuroscience Research Group (NeURos), NeuroVitae Center for Neuroscience, School of Medicine and Health Sciences, Universidad del Rosario, Bogota 111221, Colombia; 4 Fundación CardioInfantil-Instituto de Cardiología, Bogota 111156, Colombia; jrodriguez@lacardio.org; 5 Hospital Universitario Mayor Mederi, Bogota 111411, Colombia

**Keywords:** obstructive sleep apnea, GLP-1-based therapies, GLP-1 receptor agonists, tirzepatide, intermittent hypoxia, obesity, apnea–hypopnea index, metabolic dysfunction, visceral adiposity

## Abstract

Obstructive sleep apnea (OSA) affects nearly one billion adults worldwide and involves mechanical upper-airway obstruction alongside metabolic dysfunction, systemic inflammation, and gut–brain axis disruption. Despite the efficacy of continuous positive airway pressure (CPAP), suboptimal long-term adherence highlights the need for complementary pharmacological strategies. This narrative review synthesizes preclinical and clinical evidence on GLP-1-based therapies, encompassing selective GLP-1 receptor agonists and tirzepatide, a dual GIP/GLP-1 receptor agonist, in obesity-associated OSA. Preclinical studies show that GLP-1 receptor activation can reduce visceral adiposity, attenuate pro-inflammatory signaling, improve glucose and lipid metabolism, and modulate hypothalamic appetite circuits. However, the only identified murine study evaluating liraglutide during intermittent hypoxia—an experimental model of one component of OSA rather than the complete disorder—did not reverse hypoxia-induced insulin resistance, indicating that persistent intermittent hypoxia may limit metabolic improvement in that model. Clinically, liraglutide and tirzepatide have reduced the apnea–hypopnea index (AHI) and improved several cardiometabolic outcomes. Numerically larger AHI reductions were reported in the tirzepatide trials, although no head-to-head comparison with selective GLP-1 receptor agonists is available. Tirzepatide is approved by the U.S. Food and Drug Administration for moderate-to-severe OSA in adults with obesity, together with a reduced-calorie diet and increased physical activity, and may be used without concomitant positive airway pressure in eligible patients or as an adjunct to ongoing therapy. The observed respiratory benefits appear to be mediated predominantly by weight loss and related reductions in mechanical and metabolic burden. Evidence supporting additional weight-independent mechanisms remains insufficient, and longer-term mechanistic studies in broader populations are required.

## 1. Introduction

Obstructive sleep apnea (OSA) is a highly prevalent and heterogeneous sleep-related breathing disorder characterized by recurrent episodes of partial or complete upper-airway obstruction during sleep, leading to intermittent hypoxemia, sleep fragmentation, increased respiratory effort, and excessive daytime sleepiness [1,2]. Its global burden has increased in parallel with obesity, population aging, and the rising prevalence of cardiometabolic disease, with estimates suggesting that OSA affects nearly one billion adults worldwide [3]. Despite this high burden, OSA remains substantially underdiagnosed, particularly in settings with limited access to polysomnography.

Although OSA has traditionally been conceptualized as a disorder primarily driven by anatomical and mechanical upper-airway collapsibility, its pathophysiology extends beyond airway obstruction. Intermittent hypoxia, oxidative stress, recurrent arousals, sympathetic overactivation, neuroendocrine disruption, systemic inflammation, and metabolic dysfunction contribute to disease progression and to the development of distinct clinical phenotypes. These mechanisms are closely interrelated with obesity, insulin resistance, leptin resistance, cardiovascular disease, and neurological comorbidities, supporting the current view of OSA as a multisystemic disorder rather than an isolated respiratory condition [4,5,6].

Continuous positive airway pressure (CPAP) remains the standard and most extensively validated therapy for maintaining upper-airway patency during sleep [7]. CPAP reduces excessive daytime sleepiness, improves sleep-related quality of life, and may contribute to better blood pressure control and reduced accident risk [1]. However, its therapeutic effect is predominantly mechanical and does not directly address the metabolic, inflammatory, autonomic, and neuroendocrine abnormalities that frequently accompany OSA. In addition, long-term adherence remains suboptimal, with fewer than half of patients meeting conventional adherence criteria, commonly defined as use for at least 4 h per night on 70% of nights [7]. In this context, pharmacological strategies targeting obesity and metabolic dysfunction have gained increasing interest. Selective glucagon-like peptide-1 receptor agonists (GLP-1RAs) and tirzepatide, a dual glucose-dependent insulinotropic polypeptide (GIP)/GLP-1 receptor agonist, are particularly relevant because they produce clinically meaningful weight loss and improve glycemic control and cardiometabolic risk. For concision, the term “GLP-1-based therapies” is used throughout this review to encompass both selective GLP-1RAs and dual GIP/GLP-1 receptor agonism; class-specific distinctions are stated when relevant. These actions overlap with several pathophysiological domains implicated in OSA, providing a translational rationale for their potential therapeutic role. This narrative review examines the pathophysiological basis, preclinical evidence, and emerging clinical data supporting GLP-1-based therapies in OSA, with particular emphasis on metabolic, inflammatory, cardiovascular, and neuroendocrine effects.

## 2. Methods

This narrative review was informed by targeted, non-systematic searches of the biomedical literature. Because the objective was to provide a broad translational synthesis rather than to answer a narrowly defined comparative question, no formal systematic-review protocol, PRISMA-based selection process, or exhaustive search strategy was applied. Search terms included combinations of “obstructive sleep apnea”, “sleep-disordered breathing”, “GLP-1 receptor agonists”, “GLP-1 analogues”, “liraglutide”, “semaglutide”, “tirzepatide”, and “incretin-based therapies”. Depending on the topic of each section, these terms were combined with “intermittent hypoxia”, “upper airway collapsibility”, “obesity”, “visceral adiposity”, “tongue fat”, “insulin resistance”, “inflammation”, “gut–brain axis”, “leptin resistance”, “preclinical models”, “clinical trials”, and “apnea–hypopnea index”. Priority was given to original preclinical and clinical studies directly examining GLP-1-based therapies in OSA, complemented by systematic reviews, meta-analyses, major mechanistic studies, and relevant clinical guidelines. Publications were selected according to their relevance to the translational objectives of the review, methodological robustness, and contribution to the mechanistic or clinical interpretation of the available evidence.

## 3. Conceptual and Pathophysiological Framework

OSA is a complex syndrome that includes interrelated pathophysiological processes leading to neural, muscular, respiratory, and cardiovascular signs and symptoms [8,9]. From a ventilatory perspective, anatomical and neuromuscular alterations promote recurrent pharyngeal collapse, leading to upper-airway obstruction, intermittent hypoxia, and sleep fragmentation. Typical clinical manifestations include loud snoring, witnessed apneas, excessive daytime sleepiness, and impaired cognitive function; these features correlate with disease severity and progression. However, ventilatory alterations do not fully explain OSA’s heterogeneous etiology and the diversity of clinical phenotypes. Consequently, new mechanisms have been proposed to better characterize OSA pathophysiology, with the goals of prevention, earlier diagnosis, improved phenotype discrimination, and the development of targeted therapies [9,10].

In the following sections, we first describe the ‘classical’ mechanisms contributing to OSA: (1) anatomical narrowing and pharyngeal collapse; (2) obesity and regional adiposity; and (3) ventilatory control instability. These mechanisms are subsequently integrated into the contemporary physiological endotype model, which includes upper-airway collapsibility, ventilatory control instability or loop gain, respiratory arousal threshold, and upper-airway muscle responsiveness. We then review metabolic and endocrine pathways that may amplify these endotypes, including (1) insulin resistance and metabolic dysfunction; (2) brain–gut interactions and incretin signaling; and (3) alterations in glucolipid metabolism driven by intermittent hypoxia (IH).

### 3.1. ‘Classical’ Pathophysiological Mechanisms

#### 3.1.1. Anatomic and Neuromuscular Alterations: Pharyngeal Collapse

Pharyngeal collapse in OSA results from the interaction between an anatomically vulnerable upper airway, increased surrounding tissue pressure, and insufficient neuromuscular compensation. The pharynx behaves as a collapsible tube in which luminal patency depends on the balance between intraluminal and extraluminal pressures. Accordingly, reductions in transmural pressure and increases in the critical closing pressure (P_crit_) reflect a greater passive tendency of the airway to collapse [5,8,11]. In patients with OSA, P_crit_ is typically elevated, indicating increased upper-airway collapsibility.

Anatomical factors such as craniofacial restriction, posterior or inferior hyoid displacement, soft-palate enlargement, macroglossia, adenotonsillar hypertrophy, and redundant pharyngeal tissue reduce the retropalatal and retroglossal airway lumen and increase extraluminal loading. Regional contributors, including nasal obstruction, oral breathing, posterior tongue displacement, and rostral fluid shift during recumbency, may further increase peripharyngeal tissue pressure and airway collapsibility [12,13,14]. Together, these mechanisms create a mechanically unstable airway that becomes susceptible to inspiratory collapse, particularly when neuromuscular compensation is impaired.

Neuromuscular dysfunction also contributes to upper-airway instability. OSA does not necessarily reflect a simple reduction in pharyngeal dilator activity but may involve sleep-related discoordination between tongue protrusor and retractor muscles [15]. During wakefulness, coordinated activation of the genioglossus, styloglossus, and hyoglossus increases tongue stiffness and helps preserve pharyngeal patency. During obstructive events, genioglossus activity may increase in response to negative intraluminal pressure and rising respiratory drive. However, this activation does not always produce effective airway dilation. Recruitment may be delayed, insufficient, or poorly coordinated with tongue retractor muscles, limiting the mechanical effectiveness of protrusor activation and increasing pharyngeal distensibility [11,16,17,18].

**Figure 1:**
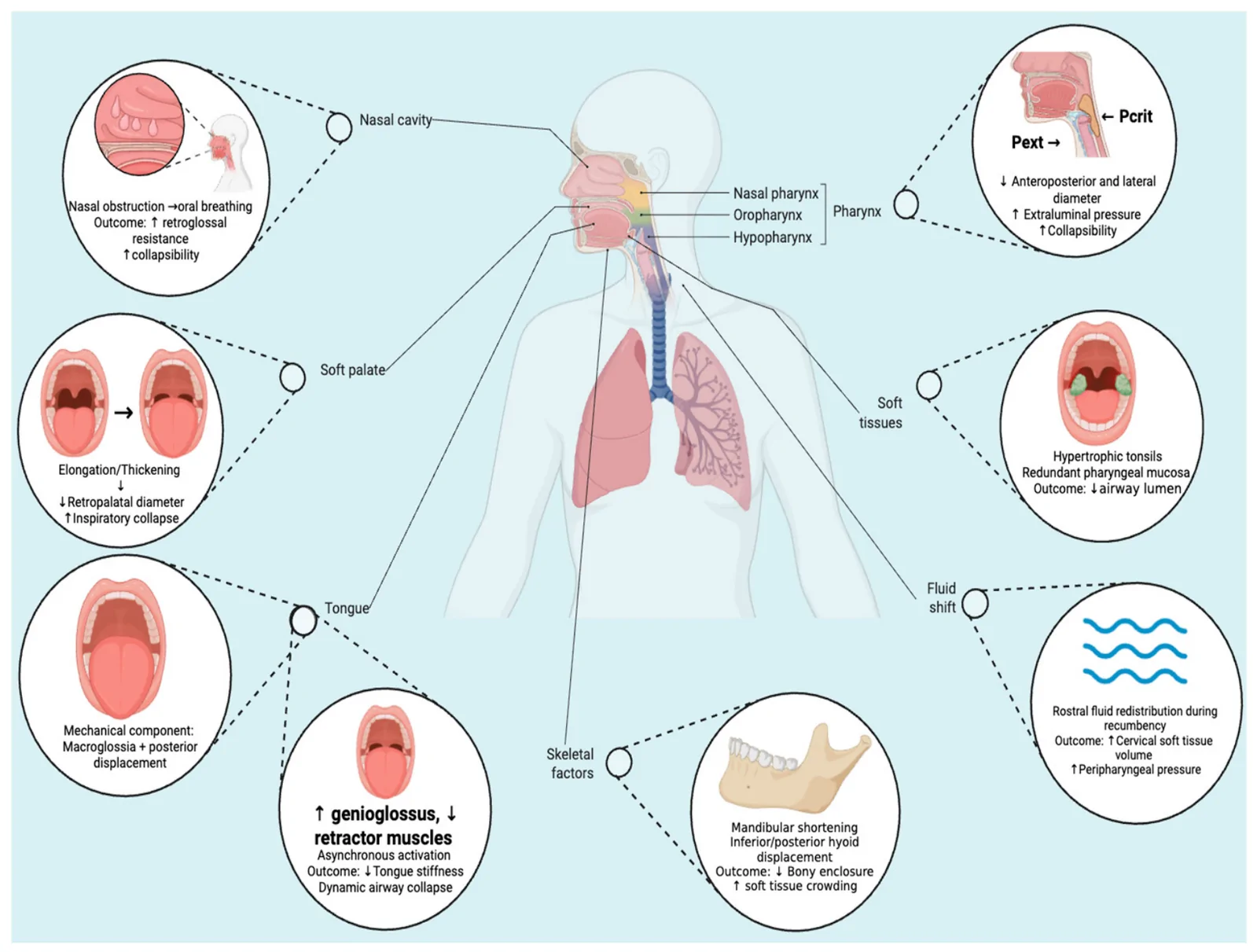
Anatomical and neuromuscular determinants of upper-airway collapsibility in OSA: Structural and functional factors involved in the upper-airway obstruction in obstructive sleep apnea. Anatomical contributing factors include nasal obstruction, soft palate elongation, tonsillar hypertrophy, macroglossia, craniofacial restriction (mandibular shortening and hyoid displacement), and rostral fluid shifts, all of which reduce airway caliber and increase extraluminal pressure. Neuromuscular dysfunction is characterized by impaired coordination between the genioglossus and pharyngeal dilator muscles, which further compromises airway stability. The interaction between these factors promotes increased upper-airway resistance and dynamic collapsibility during sleep. Arrows indicate direction of effect: ↑: increase, ↓: decrease. Created in BioRender. Gomez, I. (2026) [https://app.biorender.com/illustrations/69cfc89c3eb36a580585ff3c?slideId=3f493ce3-e05f-4dac-9375-b01e4ed530ce (accessed on 3 August 2026)].

Sleep onset further compromises airway stability by reducing central and reflex-mediated drive to the upper-airway dilator muscles. The attenuation of negative-pressure reflexes, together with rapid eye movement (REM)-related skeletal muscle atonia and ventilatory instability in individuals with high loop gain, can reduce compensatory dilator activation and promote recurrent obstruction [5,8]. These classical anatomical and neuromuscular mechanisms explain the mechanical substrate of OSA (Figure 1), but they also highlight why airway-directed interventions alone may be insufficient in patients whose disease is amplified by obesity, metabolic dysfunction, inflammation, and neuroendocrine dysregulation.

#### 3.1.2. Obesity and Fat Distribution

Obesity contributes to OSA pathophysiology not only through excess body weight but also through the regional distribution of adipose tissue. Cervical, peripharyngeal, and visceral adiposity are particularly relevant because they combine mechanical effects on upper-airway patency with systemic metabolic and inflammatory consequences. Increased visceral fat has been associated with greater cervical adiposity and upper-airway fat deposition, even among individuals with normal body mass index (BMI), suggesting that fat distribution may be more informative than BMI alone in determining OSA susceptibility [19,20]. Accordingly, anthropometric and adiposity-related indices such as neck circumference, waist-to-hip ratio, visceral adiposity index, and body fat percentage have been independently associated with OSA risk [21].

Mechanistically, regional fat accumulation promotes pharyngeal narrowing by increasing extraluminal pressure, reducing airway caliber, impairing longitudinal tracheal traction, and increasing upper-airway resistance. These effects shift transmural pressure toward collapse and contribute to an elevated critical closing pressure. Imaging studies further support the relevance of regional adiposity, showing that cervical fat tissue volume correlates with OSA severity and with polysomnographic markers such as the apnea–hypopnea index (AHI), oxygen desaturation index, and time spent below 90% oxygen saturation [22]. Sex-specific differences may also modulate this relationship, as visceral adiposity appears strongly associated with OSA in middle-aged men, whereas both visceral and subcutaneous fat distribution may be relevant in women [23].

Beyond its mechanical effects, adipose tissue acts as a metabolically active organ that may amplify OSA pathophysiology. Visceral adiposity promotes chronic low-grade inflammation and endocrine dysregulation, characterized by altered adipokine signaling, leptin resistance, reduced adiponectin, and increased pro-inflammatory mediators such as IL-6 and TNF-α [20]. These alterations may impair ventilatory control, reduce pharyngeal dilator muscle tone, and reinforce cardiometabolic dysfunction. Thus, obesity in OSA should be understood as both a structural burden and a systemic pathophysiological amplifier, linking upper-airway vulnerability with metabolic, inflammatory, and neuroendocrine dysregulation.

#### 3.1.3. Physiological Endotypes of Obstructive Sleep Apnea

The described classical mechanisms can be integrated within the contemporary endotype model of OSA. This framework recognizes that disease severity is determined by the interaction of four critical factors: increased upper-airway collapsibility, unstable ventilatory control or high loop gain, an altered respiratory arousal threshold, and insufficient upper-airway muscle responsiveness [24]. The endotype model helps explain why individuals with similar AHI values may differ in respiratory-event duration, hypoxemic burden, sleep fragmentation, clinical symptoms, cardiometabolic prognosis, and therapeutic response. This model provides a physiological basis for understanding heterogeneity in treatment response; patients with predominantly anatomical disease may benefit most from interventions that reduce structural loading or mechanically stabilize the airway [25]. In contrast, patients in whom non-anatomical traits predominate may benefit from endotype-targeted therapies aimed at reducing ventilatory instability (high loop gain), increasing the respiratory arousal threshold in carefully selected patients with a low threshold, or augmenting pharyngeal dilator muscle responsiveness [26,27].

The endotype framework is also relevant for assessing the effects of GLP-1-based therapies [28,29]. The major physiological endotypes involved in OSA, their pathophysiological relevance, contribution to disease severity, and the potential mechanisms through which GLP-1 receptor agonists may influence them are summarized in Table 1. Their most plausible mechanism is a reduction in upper-airway collapsibility secondary to weight loss and decreased mechanical loading from central and upper-airway adiposity, including reductions in tongue and peripharyngeal fat [30]. Beyond these anatomical effects, changes in loop gain, arousal threshold, and pharyngeal muscle compensation may also occur through weight-loss-related alterations in ventilatory control, leptin signaling, inflammation, autonomic regulation, and neuromuscular function [31,32,33,34]. However, direct evidence specific to GLP-1-based therapies remains preliminary. Although broader weight-loss studies have demonstrated improvements in upper-airway collapsibility and loop gain, changes in arousal threshold and muscle compensation have been inconsistent. To date, direct endotypic evidence for tirzepatide has been limited to a single-patient report describing improved upper-airway patency, reduced loop gain, and enhanced compensatory pharyngeal muscle responsiveness [32].

### 3.2. Emerging Metabolic Dimensions of OSA Pathophysiology

Recent evidence supports a shift from a purely mechanical understanding of OSA toward a broader metabolic and systemic framework. Recurrent hypoxia–reoxygenation cycles, sleep fragmentation, and sympathetic activation may promote oxidative stress, endothelial dysfunction, inflammation, and cardiometabolic impairment [32,33]. Within this model, insulin resistance, dyslipidemia, adipokine dysfunction, leptin resistance, impaired incretin signaling, and mitochondrial stress are not merely comorbid phenomena but potential contributors to disease progression and therapeutic response. This perspective provides the rationale for examining metabolic pathways, including GLP-1–mediated mechanisms, as clinically relevant targets in OSA [34,35].

#### 3.2.1. Metabolic Dysfunction: Insulin Resistance and Glycolipid Dysregulation in OSA

Substantial evidence demonstrates a robust association between OSA and metabolic dysfunction. Chronic intermittent hypoxia (CIH) and sleep fragmentation, the hallmark features of OSA, contribute significantly to insulin resistance through sympathetic overactivation, dysregulation of the hypothalamic–pituitary axis, and increased production of reactive oxygen species (ROS), all of which impair insulin signaling in peripheral tissue. Consistently, CIH is associated with reduced insulin sensitivity, impaired glucose tolerance, elevated fasting glucose levels, and an increased risk of type 2 diabetes mellitus (T2DM), even among non-obese individuals, indicating that OSA contributes to metabolic dysfunction independently of adiposity [36,37,38].

At the tissue level, adipose dysfunction represents a major mechanistic link between OSA and metabolic impairment. CIH promotes macrophage infiltration into adipose tissue and increases the secretion of pro-inflammatory cytokines, including TNF-α, IL-6, and IL-8, which disrupt insulin receptor signaling and contribute to systemic insulin resistance. Concurrent alterations in adipokine secretion, particularly elevated leptin and reduced adiponectin levels, further exacerbate metabolic dysregulation. Leptin resistance enhances sympathetic activation, whereas reduced adiponectin impairs insulin sensitivity in skeletal muscle and liver [39,40].

Several molecular pathways have been implicated in these alterations. Intermittent hypoxia induces overexpression of hypoxia-inducible factor-1 (HIF-1), which suppresses peroxisome proliferator-activated receptor gamma (PPARγ), a key regulator of GLUT-4-mediated glucose uptake, adipocyte differentiation, lipid droplet formation, and insulin sensitivity. Consequently, reduced PPARγ activity impairs glucose transport and adipose tissue function, promoting insulin resistance [41,42]. HIF-1 also activates hepatic lipogenic regulators such as sterol regulatory element-binding protein-1 (SREBP-1) and stearoyl-CoA desaturase-1 (SCD-1), enhancing the synthesis of monounsaturated fatty acids, triglycerides, and cholesterol. These alterations favor very-low-density lipoprotein (VLDL) production, hypertriglyceridemia, and hepatic steatosis, further contributing to glucolipid dysregulation in OSA [40,43].

Beyond adipose tissue dysfunction, CIH directly affects pancreatic β-cell integrity and glucose homeostasis. Experimental studies indicate that hypoxia induces mitochondrial dysfunction and oxidative stress within β-cells, reducing glucose-stimulated insulin secretion and promoting progressive β-cell failure [36]. In addition, intermittent hypoxia has been shown to decrease CD38 expression, a critical regulator of intracellular calcium mobilization through ryanodine receptor type 2, thereby further impairing glucose-induced insulin secretion [43,44]. Simultaneously, activation of the carotid body by intermittent hypoxia increases sympathetic nervous system activity and circulating catecholamine levels, promoting hepatic glucose production, lipolysis, elevated free fatty acid concentrations, and worsening peripheral insulin resistance [40,43].

These mechanisms are supported by translational evidence from experimental, cellular, and human studies demonstrating that intermittent hypoxia promotes adipose tissue inflammation, impairs insulin signaling, and reduces insulin-stimulated glucose uptake, ultimately contributing to systemic insulin resistance [39].

Collectively, these findings support the concept that intermittent hypoxia acts as a central driver of metabolic dysfunction in OSA. Through the combined effects of hypoxia, sleep fragmentation, hormonal dysregulation, and adipose tissue dysfunction, OSA establishes a self-reinforcing pathogenic cycle that exacerbates insulin resistance and increases the risk of T2DM and broader cardiometabolic complications.

#### 3.2.2. Leptin Resistance and Ventilatory Control

Metabolic disturbances are key features of OSA pathophysiology [45]. Inflammation, oxidative stress, and endothelial dysfunction, driven by intermittent hypoxia, sleep fragmentation, and sympathetic overactivation, promote insulin resistance, hyperglycemia, and dyslipidemia, further exacerbating disease severity and cardiometabolic complications. One key mediator is leptin [6]. Obesity, present in approximately 60–80% of OSA patients, is associated with leptin resistance, hyperleptinemia, and impaired signaling [46]. Leptin regulates appetite, energy homeostasis, glucose metabolism, and neuroendocrine function and modulates central respiratory control through hypothalamic nuclei and brainstem respiratory centers, enhancing ventilatory responses to hypercapnia. Peripherally, it influences ventilation through chemoreceptor interactions and respiratory muscle function [46].

Centrally, leptin acts on the dorsomedial hypothalamus and nucleus tractus solitarius, enhancing ventilatory drive and restoring neuromuscular reflexes of the upper airway [47,48,49,50]. Peripherally, it activates respiratory muscles and upper-airway dilators, reducing airway collapsibility during sleep [51]. In OSA, reduced leptin transport across the blood–brain barrier leads to relative central deficiency despite elevated circulating levels, blunting ventilatory responses, attenuating upper-airway neuromuscular activation, and contributing to hypoventilation and elevated PaCO_2_ [43,45]. This resistance is also linked to insulin resistance and altered GLP-1 incretin pathways, further impairing hypothalamic signaling and neuroendocrine feedback [52,53].

Leptin replacement can acutely improve ventilatory drive and reverse respiratory depression independently of weight loss [33,54]. The interaction between leptin and GLP-1 pathways is particularly relevant: GLP-1 receptor agonists may exert short-term metabolic benefits independently, while long-term respiratory improvements depend on intact hypothalamic leptin signaling, suggesting a synergistic therapeutic strategy [55].

#### 3.2.3. Gut–Brain Axis and Incretins

The gut–brain axis is a bidirectional network connecting the intestinal microbiota to the peripheral and central nervous systems through immune, neuroendocrine, and vagus nerve pathways, maintaining homeostasis and contributing to OSA pathophysiology. In OSA, intermittent hypoxia induces intestinal dysbiosis, compromising barrier integrity and allowing bacterial antigen translocation into the bloodstream. This activates a systemic inflammatory response, increasing Th_1_ and Th_17_ cells and TNF-α, which crosses the blood–brain barrier and induces neuroinflammation [34,56].

At the hormonal level, incretins modulate satiety and appetite, which are consistently altered in OSA [57,58]. GLP-1, with receptors in the vagus nerve, CNS, and circumventricular organs, promotes satiety and reduces gastric emptying under physiological conditions. In OSA, intermittent hypoxia and sympathetic activation disrupt incretin secretion, while sleep fragmentation reduces GLP-1 levels, decreasing satiety. Concomitant leptin resistance, elevated ghrelin, and elevated cortisol sustain chronic inflammation [34,58]. At the CNS level, the arcuate nucleus regulates hunger through NPY/AgRP and POMC/CART neuronal populations [59]. NPY is upregulated in OSA independently of BMI, directly linked to apnea rather than adiposity, increasing appetite, sympathetic tone, and cardiovascular risk [60,61,62,63,64,65]. Chronic sleep deprivation further activates reward circuits, promoting hedonic eating [57].

**Figure 2:**
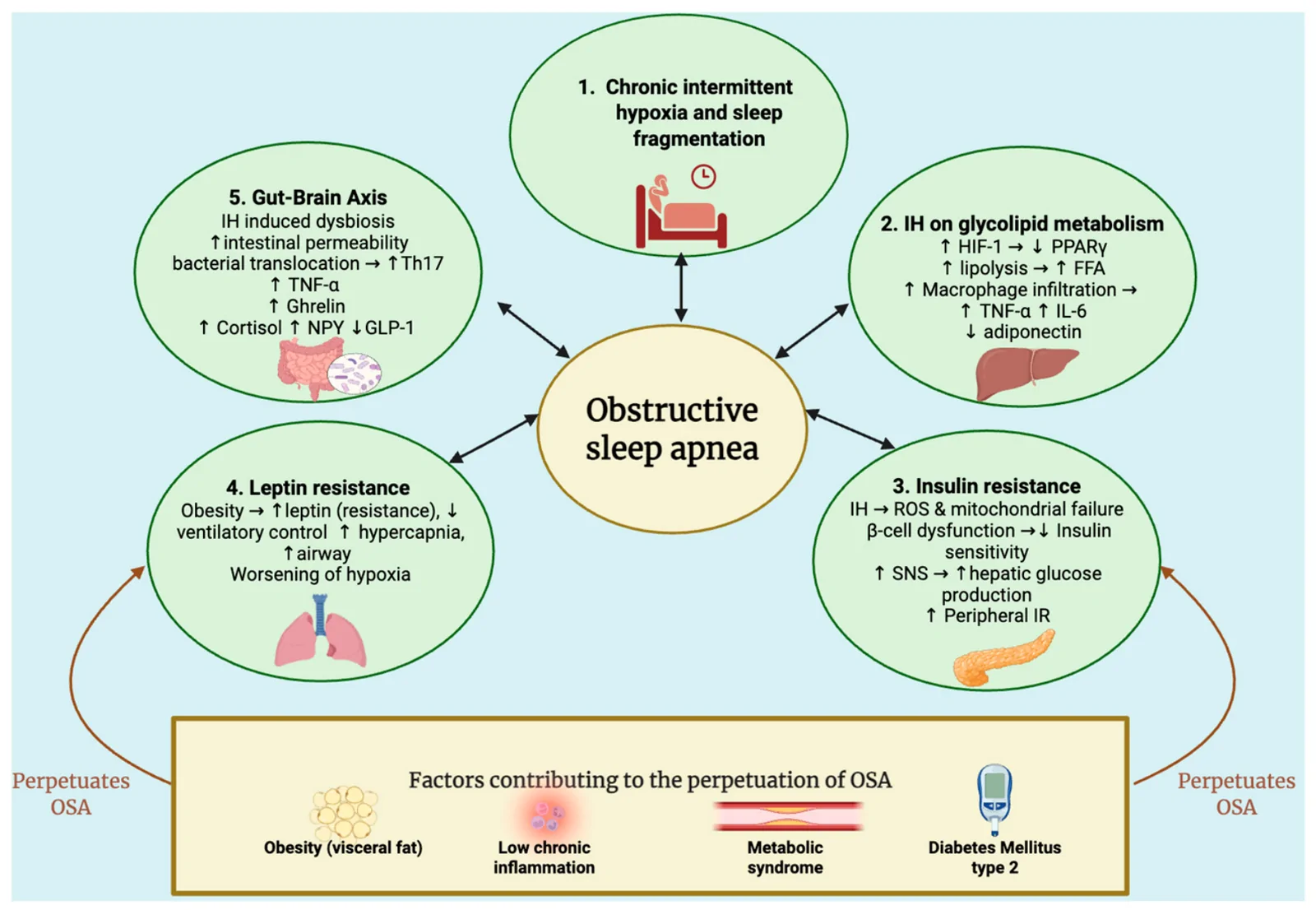
Metabolic and neuroendocrine dysregulations as key drivers of the pathogenesis and progression of obstructive sleep apnea. The primary consequence of OSA is intermittent hypoxia (IH) and sleep fragmentation, which trigger activation of the sympathetic nervous system (SNS), which consequently induces the production of reactive oxygen species (ROS), proinflammatory cytokines, and oxidative stress, promoting insulin resistance (IR) through mitochondrial dysfunction, impaired insulin signaling, and β-cell dysfunction. Activation of hypoxia-inducible factor 1 (HIF-1) inhibits peroxisome proliferator-activated receptor gamma (PPARγ) and glucose transporter type 4 (GLUT-4), promoting lipolysis, increased free fatty acids (FFAs), and dyslipidemia. Leptin has been linked to enhancing ventilatory drive and restoring protective neuromuscular reflexes of the upper airway; therefore, leptin resistance is associated with inefficient ventilatory control, increasing airway collapsibility as well as an insufficient response to hypercapnia and increased hypoxia. Furthermore, IH causes gut dysbiosis, disrupting the gut–brain axis and altering the secretion of incretins and appetite-regulating hormones—such as downregulation of glucagon-like peptide 1 (GLP-1) and upregulation of neuropeptide Y (NPY) and ghrelin—which results in increased appetite and chronic systemic inflammation. Taken together, these mechanisms converge in a bidirectional cycle that perpetuates OSA and promotes cardiometabolic complications. Arrows indicate direction of effect ↑: increase, ↓: decrease, →: leads to. Created in BioRender. Gomez, I. (2026) [https://app.biorender.com/illustrations/69f2a9440a5f941aaa7e17b1?slideId=39ef1358-0670-420d-a398-1ef616ac5c5f, (accessed on 3 August 2026)].

Collectively, obesity promotes OSA through increased pharyngeal fat and airway collapsibility, while OSA, through gut–brain axis disruption and altered incretin signaling, hinders weight loss, increases caloric intake, and sustains a pro-inflammatory state that reinforces resistance to anorexigenic incretins and heightened sensitivity to orexigenic ones (Figure 2) [58,60,61,62,63,64,65,66,67].

## 4. Mechanisms of Action of GLP-1-Based Therapies

### 4.1. Physiology of GLP-1 and Its Receptor

GLP-1R exhibits a broad tissue distribution; within the CNS, it is expressed in hypothalamic and brainstem regions such as the NTS, where it regulates anorexigenic pathways through activation of pro-opiomelanocortin (POMC) and cocaine- and amphetamine-regulated transcript (CART) neurons, inhibition of neuropeptide Y/agouti-related peptide (NPY/AgRP) neurons, and modulation of vagal afferent signaling [68].

Beyond the canonical cAMP/PKA pathway, GLP-1R activation engages several additional intracellular signaling cascades that broaden its mechanistic and therapeutic relevance. In parallel to PKA activation, the rise in cAMP levels also activates the Epac2 (exchange protein activated by cAMP) pathway [69,70]. Through Epac2 activation, GLP-1 promotes rapid Ca^2+^-dependent insulin exocytosis and mobilizes Ca^2+^ from intracellular stores via sensitization of ryanodine receptors (RYRs). The resulting increase in cytosolic and mitochondrial Ca^2+^ further stimulates ATP production, thereby enhancing glucose-dependent insulin secretion [70]. In addition, GLP-1R activation stimulates phosphatidylinositol-3-kinase (PI3K), which promotes the phosphorylation and activation of Akt (protein kinase B, PKB). In pancreatic β-cells, the PI3K/Akt pathway promotes cell survival signals through the regulation of anti-apoptotic proteins such as Bcl-2 and Bcl-xL and the inhibition of pro-apoptotic mediators such as Bax and caspase-3 while also supporting proliferation and maintenance of β-cell mass. In peripheral tissues, this signaling pathway contributes to improved insulin sensitivity and lipid metabolism [71]. Furthermore, GLP-1R activation by its analogs stimulates the mitogen-activated protein kinase (MAPK) pathway, particularly the RAF–MEK–ERK1/2 cascade, in which ARAF, BRAF, and RAF1 kinases act as upstream mediators of ERK activation. In pancreatic β-cells, this signaling pathway, in interaction with the PKA pathway, promotes cell cycle progression, supporting proliferation, maintenance of functional β-cell mass, and survival signaling in response to external stimuli and cellular stressors. Additionally, the MAPK/ERK pathway integrates with other pathways such as PI3K/Akt and Ras to coordinate cellular metabolism and function, while different GLP-1 analogs may generate differential patterns of MAPK activation, including JNK1, JNK2, and JNK3 kinases, potentially modulating the intensity and duration of insulin secretion [72]. This mechanistic diversity is particularly relevant to the pathophysiology of OSA, as it provides a plausible molecular basis for the pleiotropic effects of GLP-1RAs described throughout this review, including glycemic control, adipose tissue remodeling, and anti-inflammatory signaling, all of which converge on pathways implicated in obesity-associated OSA.

Peripherally, GLP-1R is expressed in the endocrine pancreas, kidneys, gastrointestinal tract, cardiovascular system, and vagal afferent fibers [73]. Tissue-distribution studies indicate that, within muscle, receptor expression is detected in vascular smooth muscle and cardiac tissue, whereas convincing expression has not been demonstrated in human skeletal muscle [74,75]. Consistent with this observation, no direct evidence confirms GLP-1R expression in the genioglossus or other pharyngeal skeletal muscles. Therefore, a direct receptor-mediated pharmacological effect on upper-airway neuromuscular function remains unproven, and any benefit is more plausibly mediated indirectly through weight loss, reduced mechanical loading, or metabolic improvement. In the pancreas, GLP-1R activation enhances glucose-dependent insulin secretion while suppressing glucagon release. Within the gastrointestinal tract, it delays gastric emptying and promotes satiety, thereby reducing caloric intake [73,76]. GLP-1R activation has also been associated with natriuresis, endothelial effects, modulation of autonomic signaling, inhibition of NF-κB pathways, and reductions in pro-inflammatory cytokines such as IL-6 and TNF-α [77,78,79]. However, the extent to which these effects independently improve OSA has not been established.

Building upon the mechanisms previously described, the interaction of GLP-1 with the gut–brain axis provides insight into how peripheral signals are integrated at the central level to regulate energy balance and other neuroendocrine functions. This axis constitutes a bidirectional communication system in which the intestine functions as a sensory organ capable of directly influencing brain activity through multiple signaling pathways. In this context, GLP-1 released from intestinal L cells participates in two principal mechanisms of communication with the central nervous system: a neural pathway and an endocrine pathway, which operate in a complementary manner. The neural pathway, considered the predominant mechanism, is mediated by vagal afferent fibers. GLP-1 acts on receptors expressed on vagal afferent terminals, generating signals that are transmitted to the brainstem and subsequently relayed to hypothalamic structures such as the arcuate nucleus, where responses related to satiety and the regulation of energy metabolism are integrated [66,67]. In parallel, endocrine signaling represents an additional pathway through which a fraction of circulating GLP-1 can cross the blood–brain barrier and exert direct effects on central receptors. Although its contribution is less prominent than that of vagal signaling, this pathway participates in the modulation of hypothalamic circuits involved in the regulation of appetite and metabolic homeostasis [68,69].

The integration of these pathways is potentially relevant to OSA, in which central respiratory control, metabolic regulation, and systemic inflammation interact [78,80]. Vagal GLP-1 signaling reaches brainstem and hypothalamic regions involved in autonomic and metabolic regulation, providing an anatomical rationale for possible effects on respiratory control [81]. Nevertheless, direct modulation of CO_2_ chemosensitivity, ventilatory stability, or upper-airway patency by GLP-1-based therapies has not been demonstrated in patients with OSA. GLP-1 signaling also exerts systemic anti-inflammatory effects that may be relevant to the chronic low-grade inflammation, oxidative stress, and endothelial dysfunction associated with OSA. Together with effects on body weight and energy metabolism, these observations support a biologically plausible therapeutic rationale, but they should not be interpreted as evidence of a confirmed weight-independent respiratory mechanism [82].

### 4.2. Relevant Effects in Obstructive Sleep Apnea

#### 4.2.1. Weight Loss and Fat Redistribution

Obesity is one of the principal contributors to both the development and severity of OSA. Accordingly, weight reduction represents the most extensively described therapeutic effect of GLP-1RAs and is considered one of the primary mechanisms through which these agents may improve OSA outcomes [83].

GLP-1RAs promote weight loss through coordinated central and peripheral mechanisms. At the central level, GLP-1 signaling modulates appetite and energy balance through hypothalamic and brainstem pathways. Within the arcuate nucleus, GLP-1R activation stimulates anorexigenic POMC and CART neurons while inhibiting orexigenic NPY/AgRP pathways, resulting in reduced food intake and increased energy expenditure [66,70]. In addition, GLP-1 signaling modulates mesolimbic reward circuits, particularly within the ventral tegmental area, reducing the rewarding properties of food and attenuating hedonic eating behaviors [82].

Peripherally, GLP-1R activation delays gastric emptying and reduces gastrointestinal motility, prolonging gastric distension and enhancing satiety signaling through vagal afferent pathways [84]. Furthermore, GLP-1 influences the secretion of appetite-regulating hormones by reducing ghrelin levels while increasing peptide YY and cholecystokinin, thereby further promoting satiety and decreasing caloric intake [85]. Experimental evidence, drawn from animal models (particularly rodents) used to characterize the neuronal circuits underlying appetite regulation, cellular models employed to examine molecular signaling and receptor translocation, and human studies validating the clinical impact of these hormones on satiety and glycemic control, also suggests a functional interaction between GLP-1 and leptin signaling pathways, supporting complementary effects on appetite regulation and body-weight control [86,87].

Beyond appetite regulation, GLP-1RAs improve glucose homeostasis through stimulation of glucose-dependent insulin secretion and suppression of glucagon release, leading to reduced hepatic glucose production and improved glycemic control [88,89]. These metabolic effects may further contribute to reductions in adiposity and improvements in obesity-related complications.

Collectively, GLP-1RA-induced weight loss results from the integrated modulation of gut–brain signaling, appetite-regulating neurocircuits, gastrointestinal motility, and glucose homeostasis. Given the central role of obesity in OSA pathophysiology, these mechanisms provide a strong biological rationale for the therapeutic use of GLP-1RAs in this population.

#### 4.2.2. Reduction in Fat Deposits in the Upper Respiratory Tract

As explained in the previous section, fat distribution plays an important role in the pathophysiology of OSA. Specifically, VAT has been consistently correlated with worsening of OSA severity by promoting pharyngeal collapsibility and decreased compensatory neuromuscular responses among the other mechanisms mentioned [23,90,91]. This is particularly important, as GLP-1RAs such as exenatide and liraglutide have been shown to decrease both VAT and SAT, with a more pronounced effect on VAT likely due to the higher density of GLP-1Rs in visceral fat depots. Activation of these receptors promotes adipocyte breakdown, leading to a decrease in VAT [92].

Moreover, MRI-based studies have demonstrated that liraglutide significantly reduces visceral fat while slightly increasing subcutaneous fat. The proposed mechanisms include downregulation of lipogenesis-related genes such as Mgat and Dgat2, promotion of white adipose tissue browning, and central effects previously described, including enhanced satiety through activation of POMC neurons in the hypothalamus [93].

Likewise, it has been evidenced that these agents promote a reduction in waist circumference in both obese patients without comorbidities and obese patients with DM, with a more prominent improvement in those without comorbidities, given that those with DM already present alterations in gastric and intestinal motility, explaining why they lose less visceral fat compared to non-diabetic individuals [94]. In addition to these effects on adipose tissue distribution, in one study semaglutide has been shown to significantly reduce tongue fat tissue and fat proportion in women with polyendocrine metabolic ovarian syndrome through MRI, suggesting that it may play an important role in upper-airway collapsibility and attenuation of OSA [95].

Additionally, GLP-1 has been shown to reduce ectopic fat accumulation associated with cardiometabolic syndrome, such as liver adipose tissue (LAT) and epicardial adipose tissue (EAT), not only by promoting satiety but also by direct mechanisms such as improving insulin sensitivity, attenuating inflammation, and activating brown tissue, which increases thermogenesis and promotes an increase in whole-body energy expenditure [96]. This is particularly relevant given that VAT has been shown to promote systemic inflammation, LAT promotes hepatic steatosis and insulin resistance, and EAT is associated with an increased risk of atherosclerosis, all of which are attenuated by GLP-1 intervention and consequently improve the cardiometabolic profile of patients [97,98,99].

Collectively, these findings suggest that GLP-1RAs could potentially attenuate OSA pathophysiology not only through overall weight reduction but also by selectively targeting fat depots directly implicated in upper-airway collapsibility, as well as through modulation of the cardiometabolic syndrome—particularly relevant given that OSA pathophysiology is associated with a vicious cycle in which disease-related complications and cardiometabolic disturbances mutually reinforce its clinical manifestations and underlying mechanisms.

#### 4.2.3. Weight-Independent Effects on OSA

Beyond weight reduction, GLP-1RAs exert a series of metabolic effects that may independently influence the pathophysiology of OSA, particularly through the modulation of insulin sensitivity, glucose metabolism, and lipid profile. In terms of glycemic control, GLP-1RAs have demonstrated improvement in insulin sensitivity and reduction in blood glucose through both pancreatic and extrapancreatic mechanisms. At the pancreatic level, these drugs stimulate glucose-dependent insulin secretion and suppress glucagon release, contributing to more efficient regulation of glycemic homeostasis [97,98,99].

These effects are especially relevant in patients with insulin resistance, a condition frequently associated with OSA. Complementarily, extrapancreatic effects broaden the metabolic impact of GLP-1. These include reduction in hepatic glucose production, improvement of peripheral glucose uptake in skeletal muscle, and delay of gastric emptying, which attenuates postprandial glucose peaks [100,101]. Taken together, these mechanisms contribute to reducing hyperinsulinemia and the proinflammatory metabolic state, factors that have been linked to the severity of OSA.

Additionally, GLP-1RAs exert relevant effects on lipid metabolism and energy balance. It has been described that these drugs inhibit the synthesis and secretion of chylomicrons, reduce de novo lipogenesis, and decrease hepatic production of very-low-density lipoproteins (VLDLs), resulting in an overall improvement of the lipid profile. These effects have been attributed to multiple mechanisms, including activation of the GLP-1R at the intestinal and hepatic levels, which reduces apolipoprotein B48 expression and limits chylomicron assembly; modulation of intracellular pathways such as AMPK (AMP-activated protein kinase), which inhibits hepatic lipogenesis; and improvement of insulin sensitivity, which decreases the flow of free fatty acids to the liver and, consequently, VLDL production [102,103].

It has been demonstrated that GLP-1RAs promote adipose tissue remodeling and the browning of white adipose tissue, particularly in subcutaneous depots, through the activation of SIRT1 (sirtuin 1)-dependent pathways. Specifically, activation of SIRT1, an NAD^+^-dependent deacetylase, induces the deacetylation and activation of transcriptional coactivators such as PGC-1α (peroxisome proliferator-activated receptor gamma coactivator 1-alpha), which increases the expression of thermogenic genes, including UCP1 (uncoupling protein 1), favoring the conversion of white adipocytes into beige adipocytes with greater oxidative and thermogenic capacity [104,105]. Additionally, signaling mediated by the GLP-1R can modulate sympathetic activity and pathways such as AMPK, contributing to this metabolically active phenotype. This process results in adipose tissue with greater capacity for fatty acid oxidation and lower secretion of proinflammatory cytokines, which translates into improved insulin sensitivity and reduced systemic inflammation. In the context of OSA, these effects are particularly relevant, as adipose tissue dysfunction and chronic low-grade inflammation are key components in its pathophysiology. Thus, browning induced by GLP-1RAs could contribute to mitigating these mechanisms, independently of weight loss, supporting their potential therapeutic benefit in patients with OSA [79,106].

This process favors a metabolically more active phenotype, with greater thermogenic capacity and a lower inflammatory profile. In OSA, these metabolic effects could influence insulin resistance, systemic inflammation, and adipose-tissue dysfunction. However, most supporting studies were conducted in cellular systems, animal models, or human populations without OSA. Consequently, these pathways should be regarded as biologically plausible and hypothesis-generating rather than as established weight-independent mechanisms of AHI improvement.

#### 4.2.4. Anti-Inflammatory Effects of GLP-1

Although the metabolic benefits of GLP-1RAs are largely attributed to weight loss, improved lipid metabolism, and glycemic control, increasing evidence indicates that these agents also exert anti-inflammatory effects through both indirect metabolic mechanisms and direct actions on immune cells [107].

Indirect anti-inflammatory effects are closely linked to improvements in metabolic dysfunction. Several GLP-1RAs, including exenatide, liraglutide, semaglutide, and tirzepatide, have been shown to reduce circulating C-reactive protein (CRP) levels in patients with obesity and/or T2DM [108]. These effects are likely mediated by reductions in visceral adiposity and improvements in glycemic control, both of which attenuate the chronic low-grade inflammatory state characteristic of obesity and insulin resistance. Consistent with this mechanism, GLP-1 therapy has been associated with decreased secretion of pro-inflammatory mediators such as TNF-α, IL-6, and resistin, increased adiponectin levels, and improved insulin sensitivity in adipose tissue and skeletal muscle. Furthermore, preclinical and clinical evidence has shown GLP-1RAs may improve insulin signaling through modulation of the JNK pathway, thereby counteracting endoplasmic reticulum stress-induced insulin resistance [54,77]. Collectively, these findings suggest that GLP-1RAs attenuate chronic inflammation by targeting key metabolic drivers of immune activation.

In addition to these indirect effects, GLP-1RAs exert direct immunomodulatory actions. GLP-1R transcripts have been identified in multiple human immune-cell populations, including CD4+ and CD8+ T lymphocytes, B lymphocytes, and regulatory T cells (Tregs). Receptor activation increases intracellular cAMP, a signaling pathway associated with immunosuppressive and anti-inflammatory responses [109]. In lymphocytes, GLP-1R activation inhibits proximal T-cell receptor signaling and reduces the transcription of pro-inflammatory cytokines such as IFN-γ, TNF-α, and IL-2 [110,111]. Additional evidence from monocyte and peripheral blood mononuclear cell models demonstrates that GLP-1RAs suppress inflammatory signaling through inhibition of NF-κB activation; reduction in ROS generation; and decreased expression of inflammatory mediators, including TLR4 (Toll-like receptor 4), NLRP3 (NLR family pyrin domain containing 3), IL-1β, TNF-α, and JNK-1 [112,113,114]. Likewise, studies in human pancreatic islets have shown reductions in IL-1β, IL-2, IL-17, and IFN-γ following exenatide treatment [115].

Taken together, GLP-1-based therapies may regulate systemic inflammation through indirect metabolic improvement and direct immunomodulatory signaling. Human OSA trials have measured inflammatory biomarkers: hsCRP was included among the secondary outcomes in both the SCALE Sleep Apnea and SURMOUNT-OSA programs, and tirzepatide produced significant reductions in hsCRP in SURMOUNT-OSA [30,116,117]. These findings demonstrate improvement in an inflammatory biomarker but do not establish that suppression of systemic inflammation independently mediates the reduction in AHI. The relative contributions of improvement in nocturnal hypoxemia and possible direct anti-inflammatory actions therefore remain uncertain.

**Figure 3:**
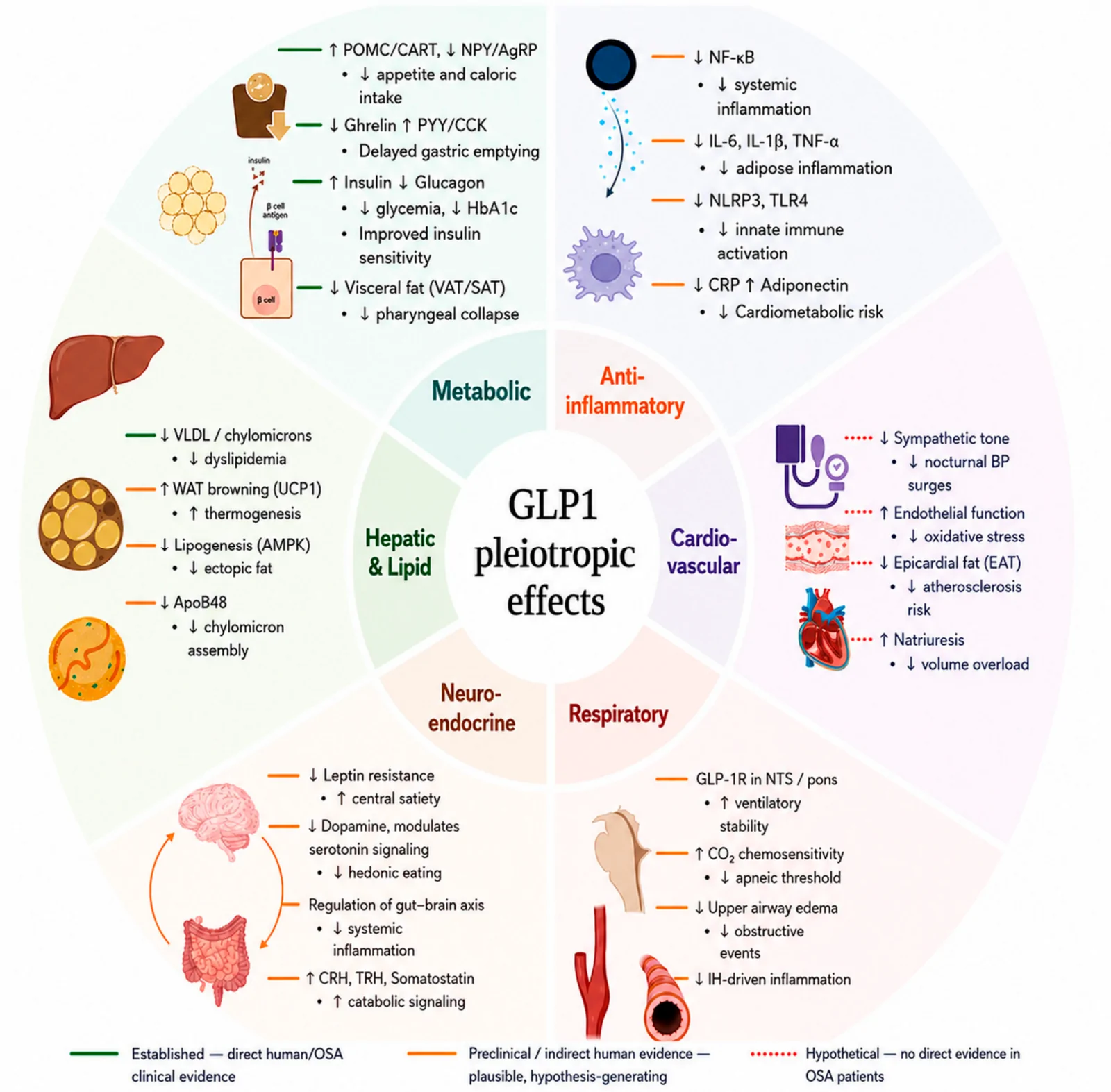
Conceptual pleiotropic pathways through which GLP-1-based therapies may influence drivers and consequences of obstructive sleep apnea. The figure integrates metabolic, inflammatory, cardiovascular, neuroendocrine, hepatic, and potential respiratory pathways described in experimental and clinical literature. To reflect differences in the strength and directness of the underlying evidence, pathways are coded by line style and color: solid green lines denote mechanisms supported by direct clinical evidence in OSA (reductions in body weight, AHI, hypoxic burden, blood pressure, and selected inflammatory biomarkers, particularly hsCRP [30,118]); solid orange lines denote mechanisms supported by preclinical, indirect human, or non-OSA-specific evidence that remain biologically plausible but hypothesis-generating; and dotted red lines denote mechanisms that are predominantly hypothetical, with no direct confirmatory evidence in patients with OSA. On this basis, restoration of leptin signaling, autonomic modulation, reduction in oxidative stress, direct effects on CO_2_ chemosensitivity or ventilatory stability, and upper-airway neuromuscular actions are coded as preclinical/indirect or hypothetical rather than established. Accordingly, the respiratory and neuroendocrine components of the figure should be interpreted as a conceptual framework rather than as established weight-independent mechanisms. Created in BioRender. Gomez, I. (2026) [https://app.biorender.com/illustrations/6a0bbe08ba0c4993d9e14570?slideId=e6c94ecb-4226-4007-8d49-34cb4a07072a, (accessed on 3 August 2026)]. Arrows indicate direction of effect (↑ increase, ↓ decrease). NTS: nucleus tractus solitarii; VAT: visceral adipose tissue; SAT: subcutaneous adipose tissue; EAT: epicardial adipose tissue; WAT: white adipose tissue; UCP1: uncoupling protein 1; VLDL: very-low-density lipoprotein; AMPK: AMP-activated protein kinase; NF-κB: nuclear factor kappa B; NLRP3: NOD-like receptor protein 3; TLR4: toll-like receptor 4; CRP: C-reactive protein; CRH: corticotropin-releasing hormone; TRH: thyrotropin-releasing hormone; IH: intermittent hypoxia.

The proposed mechanisms through which GLP-1-based therapies may influence OSA differ substantially in the type, directness, and OSA-specificity of their supporting evidence. Weight loss and the associated reduction in mechanical loading currently represent the most strongly supported explanation for improvement in OSA severity. By contrast, reductions in regional adiposity, systemic inflammation, and metabolic dysfunction are supported by varying combinations of preclinical, indirect human, and limited OSA-specific evidence, whereas effects involving oxidative stress, leptin signaling, autonomic regulation, central respiratory control, and upper-airway neuromuscular function remain predominantly hypothetical in patients with OSA. Importantly, concurrent improvements in AHI and metabolic or inflammatory variables should not be interpreted as evidence of mechanistic mediation unless this relationship has been formally evaluated. To visually synthesize these findings, Figure 3 presents a conceptual overview of the proposed pathways stratified by strength of evidence, while Table 2 provides a detailed, mechanism-by-mechanism summary of the source and OSA-specificity of the supporting data.

## 5. Preclinical Evidence for GLP-1-Based Therapies in OSA

### 5.1. Effects of GLP-1-Based Therapies on Complications Related to Intermittent Hypoxia

As described in previous sections, OSA severity is influenced by intermittent hypoxia, obesity, visceral adiposity, chronic systemic inflammation, insulin resistance, and broader metabolic dysfunction [21,44]. To the best of our knowledge, only one preclinical study has evaluated a GLP-1RA in an intermittent-hypoxia model representing one component of OSA pathophysiology [123]. O’Donnell et al. investigated liraglutide-induced weight loss and metabolic responses during intermittent hypoxia in obese C57BL/6 mice. Fifteen-week-old male mice were fed a 60% high-fat diet and randomized to intermittent hypoxia or intermittent air; during the 6-week protocol, they received liraglutide (300 μg/kg) or phosphate-buffered saline by daily subcutaneous injection. Liraglutide attenuated intermittent-hypoxia-induced IL-6 and IL-1β mRNA expression (*p* < 0.01) and reduced body weight and fasting glucose but did not significantly reduce fasting insulin or reverse intermittent-hypoxia-induced insulin resistance. These findings indicate that anti-inflammatory transcriptional and weight-related effects were insufficient to restore insulin sensitivity while hypoxic exposure continued in this experimental model.

The authors proposed that intermittent hypoxia can act as a strong driver of metabolic dysfunction through adipose-tissue inflammation and impaired insulin signaling, potentially attenuating the metabolic benefits of liraglutide while hypoxic stress persists [123]. This finding should not be interpreted as evidence that GLP-1-based therapy is ineffective for clinical OSA because the model did not reproduce recurrent upper-airway collapse, sleep fragmentation, intrathoracic-pressure swings, or the complete human syndrome. Rather, it suggests that metabolic improvement may be incomplete when intermittent hypoxia is not simultaneously relieved and supports evaluating pharmacotherapy together with airway-directed treatment when clinically indicated.

### 5.2. Indirect Mechanisms of GLP-1 Receptor Agonists on OSA Pathophysiological Drivers

#### 5.2.1. Weight Loss and Fat Redistribution

To the best of our knowledge, preclinical models have not specifically evaluated anthropometric parameters such as cervical adiposity. Nevertheless, existing evidence consistently demonstrates that GLP-1RAs reduce total fat mass through weight loss, which in turn contributes to a decrease in VAT and a more favorable redistribution of body fat. For instance, Zhao et al. utilized a murine model consisting of Goto–Kakizaki (GK) rats, a spontaneous model of T2DM, alongside Wistar rats as controls, to evaluate whether liraglutide could promote redistribution of body fat through regulation of lipid metabolism across different adipose depots [79]. After 12 weeks of daily liraglutide administration, the body fat-to-total mass ratio was significantly reduced in the intervention groups (22.40 ± 2.12%, *p* < 0.05 for 400 µg/kg/day; 21.78 ± 2.08%, *p* < 0.05 for 1200 µg/kg/day; and 24.36 ± 2.02%, *p* < 0.05 for diet control) compared to control groups (52.10 ± 1.64% in Wistar and 38.36 ± 3.36% in GK rats receiving normal saline). Additionally, total fat mass was markedly reduced in the intervention groups (85.4 ± 5.77 g for 400 µg/kg/day; 78.74 ± 11.60 g for 1200 µg/kg/day; and 89.96 ± 4.05 g for diet-control) relative to controls (256.1 ± 5.72 g in Wistar and 158.86 ± 15.31 g in GK rats treated with normal saline). Importantly, liraglutide administration was also associated with decreased visceral fat accumulation alongside an increase in subcutaneous fat deposition. This observation aligns with evidence indicating that GLP-1RAs significantly reduce both absolute and relative fat mass while promoting a more favorable redistribution of adipose tissue, which may, in turn, attenuate the pathophysiological mechanisms underlying OSA.

Additionally, weight loss per se has been associated with an attenuation of OSA severity, an effect for which GLP-1RA therapy has demonstrated significant efficacy [20]. Across multiple preclinical studies, diverse mechanisms have been proposed through which GLP-1RAs promote weight loss. Among these, Sonne et al. analyzed a mouse model with a humanized GLP-1R (hGLP-1R) and demonstrated that treatment with semaglutide for less than four weeks reduced body weight in both WT (−18.0 ± 2.0%) and hGLP-1R mice (−8.1 ± 1.7%, *p* < 0.001), although the effect was attenuated in the latter group (*p* < 0.01) [128]. The authors further identified regulation of appetite and energy balance through modulation of neuronal circuits in the central amygdala (CeA), parabrachial nucleus (PB), and NTS, together with delayed gastric emptying, as potential mechanisms contributing to these effects.

Similarly, Hayes et al. compared the food intake- and body weight-suppressive effects of liraglutide and exendin-4 in both non-obese and diet-induced obesity (DIO) rats [129]. Their findings demonstrated that body weight suppression was dose-dependent, with greater reductions observed at higher liraglutide exposures and with exendin-4 treatment. Additionally, both agents significantly reduced food intake relative to saline-treated controls over the treatment period, supporting appetite suppression as a key mechanism underlying GLP-1RA-induced weight loss.

Furthermore, Secher et al. investigated the long-term effects of liraglutide on the expression of key appetite-regulating genes within the arcuate nucleus (ARC) [130]. In DIO rats treated for 28 days with liraglutide (200 μg/kg, twice daily), a significant reduction in both body weight (*p* < 0.001) and food intake (*p* < 0.0001) was observed. At the molecular level, liraglutide increased mRNA expression of the anorexigenic peptide CART by 1.6-fold (*p* < 0.001) and prevented the compensatory increase in mRNA levels of NPY and AgRP, which typically occurs following weight loss. These findings suggest that sustained weight reduction could be mediated through direct modulation of hypothalamic appetite-regulatory circuits. Overall, these effects further contribute to appetite suppression and may help counteract mechanisms associated with OSA pathophysiology, where dysregulation of these pathways has been previously described.

#### 5.2.2. Anti-Inflammatory Effects of GLP-1 Involved in OSA

Experimental evidence indicates that IH promotes inflammatory remodeling of VAT, characterized by macrophage accumulation and activation, supporting adipose tissue inflammation as a potential therapeutic target in OSA [131].

Several authors have highlighted the anti-inflammatory effects of GLP-1, which may contribute to attenuation of this inflammatory response in OSA. For example, Zhou et al. employed a DIO mouse model to investigate the anti-inflammatory effects of a novel long-acting GLP-1RA, specifically through the inhibition of leptin expression [124]. Following 14 days of subcutaneous administration of 1.8 mg/kg of the drug every 6 days, a significant reduction in mRNA levels of TNF-α, IL-1β, IL-6, and monocyte chemoattractant protein-1 (MCP-1) was observed in DIO mice (*p* < 0.001 versus the normal saline group). Furthermore, mRNA levels of IL-4 (*p* < 0.05) and IL-13 (*p* < 0.01) were significantly elevated in treated mice compared to saline controls. This finding is particularly relevant, as these cytokines are implicated in the pathophysiology of OSA, suggesting that GLP-1 not only attenuates pro-inflammatory responses but also promotes anti-inflammatory cytokine expression.

This anti-inflammatory effect of GLP-1 is further supported by Xian et al., who utilized a male DIO mouse model to examine the effects of exenatide on inflammation, hypoxia, and angiogenesis [125]. By administering exenatide at a dose of 24 nmol/kg daily, they demonstrated that, after four weeks of treatment, exenatide significantly reduced serum levels of pro-inflammatory markers, including IL-6, plasminogen activator inhibitor-1 (PAI-1), and TNF-α (*p* < 0.05). In addition, there was a significant downregulation in the mRNA expression of inflammatory genes in epididymal adipose tissue, including IL-6, IL-1β, Mcp-1, and the macrophage marker F4/80. The authors further reported that, although obesity is associated with increased protein expression of HIF-1α in adipose tissue, exenatide treatment significantly reduced HIF-1α levels (*p* < 0.05). Moreover, hypoxia was attenuated through promotion of angiogenesis via upregulation of pro-angiogenic factors such as vascular endothelial growth factor (VEGF) and VEGF receptor 2 (VEGFR2) (*p* < 0.05). Collectively, these findings suggest that GLP-1 may attenuate both inflammatory and hypoxia-related pathways that contribute to metabolic dysfunction.

Another proposed anti-inflammatory mechanism of GLP-1 involves its effects on adipose tissue macrophages (ATMs). Lee et al. investigated the effects of GLP-1 on ATMs using both gene therapy (recombinant adenovirus–GLP-1 [rAd-GLP-1]) and pharmacological agonism (exendin-4) in murine models, including obese and diabetic mice, as well as in vitro adipocyte and macrophage systems [126]. Treatment with rAd-GLP-1 significantly decreased macrophage infiltration in epididymal adipose tissue and reduced M1-specific markers (F4/80 and Toll-like receptor 4 [TLR4]) (*p* < 0.05), as well as pro-inflammatory cytokines, including IL-6 (*p* < 0.05), TNF-α (*p* < 0.005), and MCP-1 (*p* < 0.05), whereas M2 markers remained unchanged. Additionally, rAd-GLP-1 reduced de novo lipogenesis, as evidenced by decreased mRNA expression of Srebp1c, Acc1, and Fas. Together, these findings support a role for GLP-1 in regulating adipose tissue inflammation and lipid metabolism. Similar reductions in ATM infiltration and pro-inflammatory markers were reported by Zhou et al., further supporting the ability of GLP-1 signaling to modulate adipose tissue inflammation [124].

Similarly, El Bekay et al. investigated the effects of both in vitro and in vivo administration of GLP-1 and its analogs on adipose tissue and adipocyte function, demonstrating reduced expression of lipogenic genes, including fatty acid-binding protein 4 (FABP4), SREBP1, PPARγ, lipoprotein lipase (LPL), and fatty acid synthase (FASN) in human adipocytes and explants obtained from obese patients (*p* < 0.05) [127]. This observation is particularly relevant because, as mentioned in previous sections, IH has been associated with activation of lipogenic pathways involving HIF-1, SREBP-1, and stearoyl-CoA desaturase-1 (SCD-1). Therefore, the reduction in adipogenic and lipogenic gene expression observed with GLP-1 treatment may indirectly counteract metabolic pathways implicated in OSA-related dyslipidemia [112].

Collectively, these findings suggest that GLP-1 signaling may attenuate adipose tissue inflammation and lipid dysregulation, processes that overlap with metabolic pathways activated by IH in OSA. However, these observations derive from obesity and metabolic disease models rather than OSA or IH models directly and therefore provide mechanistic support—but not direct evidence—for the role of GLP-1RA-based therapies in modulating OSA-associated metabolic dysfunction.

#### 5.2.3. Metabolic Dysfunction Improvement

As described in previous sections, OSA is associated with multiple metabolic dysfunctions arising from IH, obesity, and chronic inflammation. Although several studies have not directly evaluated the effects of GLP-1RAs in OSA, they have characterized their impact on metabolic disturbances that are also observed in this condition.

For instance, Mao et al. investigated the effects of semaglutide on liver injury and gut microbiota in a murine model of inherited obesity and non-alcoholic fatty liver disease (NAFLD) [132]. After 16 weeks of treatment (0.22 mg/kg), semaglutide significantly reduced alanine aminotransferase (ALT) (*p* < 0.0001), aspartate aminotransferase (AST) (*p* < 0.0001), the ALT/AST ratio (*p* < 0.0001), total cholesterol (TC), triglycerides (TGs) (*p* < 0.0001), and hepatic lipid deposition. In addition, it improved glucose tolerance, reduced blood glucose levels (*p* < 0.0001), and increased insulin concentrations (*p* < 0.01). The study also demonstrated increased gut microbiota alpha diversity (*p* = 0.03), accompanied by higher abundance of Alloprevotella and Alistipes and lower abundance of Ligilactobacillus and Lactobacillus.

Similarly, Madsen et al. examined liraglutide (0.2 mg/kg) and a dual GLP-1/GLP-2 receptor agonist (GUB09-145; 0.04 mg/kg) in DIO mice [133]. After four weeks of treatment, Shannon diversity increased significantly in both liraglutide-treated (*p* = 0.02) and GUB09-145-treated mice (*p* = 0.009). Both compounds also reduced plasma TC (*p* < 0.001), improved glucose tolerance, and lowered fasting blood glucose levels (*p* < 0.001).

Zhao et al. utilized GK rats and Wistar controls to evaluate the metabolic effects of liraglutide [79]. After 12 weeks, high-dose liraglutide (1200 µg/kg/day) promoted browning of subcutaneous white adipose tissue, with increased expression of Ucp1, Pgc1α, and Bmp4 (*p* < 0.05), while reducing adipocyte size across white adipose depots. TGs, TC, and non-esterified fatty acids (NEFAs) were also significantly reduced (*p* < 0.05), indicating improved lipid metabolism.

Collectively, these findings suggest that GLP-1RAs improve glucose metabolism, lipid handling, adipose tissue remodeling, and gut microbiota composition in preclinical models. However, because these studies were not conducted in dedicated OSA or IH models, they should be interpreted as mechanistic evidence supporting the potential relevance of GLP-1RAs in OSA-associated metabolic dysfunction rather than direct evidence of improvement in airway obstruction or hypoxic burden.

#### 5.2.4. Limitations of Preclinical Direct OSA Evidence

Most available preclinical studies should be considered mechanistic approximations rather than true models of OSA. Experimental work has generally isolated selected components of OSA pathophysiology—particularly intermittent hypoxia, adipose-tissue inflammation, insulin resistance, and obesity-related metabolic dysfunction—without reproducing recurrent upper-airway collapse, negative intrathoracic-pressure swings, sleep fragmentation, nocturnal arousals, hypercapnia, state-dependent pharyngeal neuromuscular control, and variable cervical or visceral adiposity [133]. As of July 2026, no direct preclinical evidence has been identified demonstrating that GLP-1 receptor agonists (mainly semaglutide, liraglutide, exenatide/exendin-4, or tirzepatide) modify any of the major physiological endotypes underlying the primary pathophysiological processes in OSA described in Section 2. Specifically, no animal study has quantified passive or active pharyngeal critical closing pressure (P_crit_) before and after treatment with these agents. Therefore, there is no direct preclinical evidence demonstrating that GLP-1-based therapies reduce upper-airway collapsibility independently of their effects on body weight or adiposity. Similarly, no preclinical investigation has measured loop gain following GLP-1 receptor agonist treatment; consequently, a direct effect on ventilatory control stability remains unproven. No animal study has evaluated the respiratory arousal threshold during GLP-1-based treatment, and this integrated sleep-state trait cannot be assessed using isolated cellular systems. Finally, no study has directly measured genioglossus electromyographic activity or pressure-dependent upper-airway dilator muscle responsiveness following administration of GLP-1 agonists. Thus, no direct preclinical evidence currently exists demonstrating an effect of these agents on P_crit_, loop gain, arousal threshold, or genioglossus responsiveness.

This limitation is particularly relevant when evaluating GLP-1 receptor agonists, whose proposed benefits in OSA are largely mediated through appetite suppression, weight loss, visceral fat reduction, and adipose tissue remodeling. However, these effects cannot be fully assessed in models that lack human-like upper-airway anatomy or do not quantify clinically relevant variables such as neck circumference, peripharyngeal fat deposition, BMI, or waist circumference. Consequently, although preclinical studies provide valuable mechanistic insights, they do not fully capture the anatomical, metabolic, and sleep-related complexity of human OSA.

Accordingly, current preclinical evidence supports the biological plausibility of GLP-1 receptor agonists in OSA rather than definitive proof of a direct disease-modifying effect. Available experimental evidence instead derives predominantly from models of intermittent hypoxia, diet-induced obesity, diabetes, adipose-tissue dysfunction, or isolated-cell exposure. These models have primarily assessed body weight, food intake, glucose and insulin homeostasis, inflammatory signaling, lipid metabolism, adipose-tissue remodeling, and end-organ injury. Intermittent-hypoxia systems reproduce cyclical oxygen desaturation but do not reproduce the initiating sleep-dependent pharyngeal obstruction, associated respiratory-effort and intrathoracic-pressure changes, cortical arousals, or state-dependent upper-airway neuromuscular responses that characterize human OSA. Consequently, favorable effects on weight, inflammation, or metabolic variables in these models cannot be interpreted as direct evidence that GLP-1-based therapies alleviate upper-airway obstruction or correct the physiological endotypes responsible for OSA.

The available studies suggest that GLP-1-based therapies may attenuate obesity-related and metabolic components of OSA pathophysiology, including visceral adiposity, inflammation, dyslipidemia, and insulin resistance. Nevertheless, existing models are insufficient to determine whether these agents directly modify sleep-dependent upper-airway obstruction or hypoxic burden. This limitation represents a translational evidence gap rather than evidence that the therapies cannot influence the major OSA endotypes. Future studies should use models that combine obesity with spontaneous or experimentally imposed sleep-dependent upper-airway obstruction and incorporate polysomnography, pressure–flow assessment of P_crit_, measurements of loop gain and ventilatory control, determination of respiratory arousal threshold, and genioglossus electromyography.

## 6. Clinical Evidence for GLP-1-Based Therapies in OSA

Clinical evidence for GLP-1-based therapies in OSA remains limited. The principal randomized evidence comprises the SCALE Sleep Apnea trial of liraglutide [118], the two phase 3 SURMOUNT-OSA trials of tirzepatide reported in a single publication [30], and two smaller randomized studies of liraglutide [119,120]. Additional publications include ancillary analyses, patient-reported outcomes from the SURMOUNT-OSA program, systematic reviews, meta-analyses, and case reports [121,122,134,135,136,137,138,139]. Participants have been predominantly male adults with moderate-to-severe OSA and obesity and/or T2DM. Trial-specific exclusion criteria included central or mixed sleep apnea, major craniofacial abnormalities, pregnancy, selected unstable cardiovascular or systemic diseases, and contraindications to the study drugs [30,118,119,120]. None of the randomized trials performed sufficiently powered prespecified interaction analyses to identify reliable physiological or anatomical predictors of treatment response. Consequently, it remains uncertain whether severe visceral adiposity, increased tongue fat, metabolic syndrome, or residual OSA during CPAP identifies patients with greater treatment benefit.

Tirzepatide is a dual GIP/GLP-1 receptor agonist rather than a selective GLP-1RA. Its principal OSA evidence derives from the two SURMOUNT-OSA trials [30], with additional patient-reported outcome analyses from the same program [134] and case-level observations [139]. In SURMOUNT-OSA, tirzepatide was initiated at 2.5 mg once weekly and increased by 2.5 mg every 4 weeks to a maximum tolerated dose of 10 or 15 mg once weekly.

Liraglutide, a selective GLP-1RA, has been evaluated in the SCALE Sleep Apnea trial and two smaller randomized studies [118,119,120], with an ancillary visceral-adipose-tissue analysis from the O’Donnell cohort [122]. Liraglutide was administered once daily, starting at 0.6 mg and increasing by 0.6 mg at weekly intervals to study-specific target doses ranging from 1.2 to 3.0 mg daily.

Lifestyle intervention, including a reduced-calorie diet and increased physical activity, accompanied pharmacotherapy in the major obesity-focused trials [30,118,119]. CPAP use varied by study: SURMOUNT-OSA included one trial in participants not receiving PAP and a second trial in participants continuing PAP, whereas the smaller liraglutide studies evaluated liraglutide alone, CPAP, or their combination [30,119,120].

### 6.1. Results

Change in AHI was the primary respiratory outcome in the principal randomized trials, whereas ancillary analyses and case reports evaluated additional physiological, metabolic, or patient-reported outcomes (Table 3). Treatment response has been reported using absolute AHI change, post-treatment AHI severity thresholds, and, in some OSA intervention literature, a reduction of at least 50% from baseline; no single response definition is universally applicable across all interventions [140,141]. Most randomized studies demonstrated a statistically significant reduction in AHI with GLP-1-based treatment. In the O’Donnell study, however, CPAP alone and CPAP combined with liraglutide produced substantially larger AHI reductions than liraglutide alone [119]. SURMOUNT-OSA also documented improvements in hypoxic burden and sleep-related patient-reported outcomes after 52 weeks of tirzepatide treatment [30,134].

Weight loss and cardiometabolic outcomes were also evaluated. A meta-analysis reported mean reductions of 10.99 kg in body weight and 1.60 kg/m^2^ in BMI with GLP-1-based therapies [121]. Altobaishat et al. reported a pooled 12.71% reduction in body weight and a 4.93 mmHg reduction in systolic blood pressure [137]. Another meta-analysis estimated mean reductions of 4.81 mmHg in systolic and 0.32 mmHg in diastolic blood pressure [121]. Tirzepatide produced numerically larger weight and AHI reductions than liraglutide across separate trials and pooled analyses, but these indirect comparisons do not establish pharmacological superiority because the studies differed in design, duration, population, and baseline disease severity [30,118,121,137]. Together, these findings support an additional cardiometabolic benefit that may contribute indirectly to improvement in obesity-associated OSA.

### 6.2. Adverse Reactions

The most frequent adverse reactions were gastrointestinal, including nausea, vomiting, diarrhea, constipation, dyspepsia, and gastroesophageal reflux symptoms. In randomized trials, these events occurred mainly during dose escalation and were generally mild to moderate [30,118,119,120]. Systematic reviews and meta-analyses similarly identify gastrointestinal tolerability as the principal limitation to dose optimization, while indicating that most events are transient and manageable with gradual escalation and clinical monitoring [121,135,136,137,138].

Serious adverse event rates were not significantly different between the active-treatment and control groups in the available OSA trials and pooled analyses [30,135,137]. In SURMOUNT-OSA, two adjudication-confirmed cases of acute pancreatitis occurred in tirzepatide-treated participants [30]. In the meta-analysis by Yang et al., non-serious adverse events were more frequent with GLP-1RAs than with control (RR 1.35, 95% CI 1.13–1.62, *p* = 0.001, I^2^ = 12%), whereas adverse events leading to discontinuation and serious adverse events were comparable between groups [135].

The current clinical evidence supports improvements in OSA severity with GLP-1 therapies. However, no clinical study to date has demonstrated a direct pharmacologic effect independent of weight loss. Available studies have not performed formal mediation analyses, established that AHI improvements precede weight reduction, or evaluated physiologic endotypes of OSA such as upper-airway collapsibility, loop gain, arousal threshold, or genioglossus muscle activity. The available clinical evidence suggests that GLP-1 receptor agonists, liraglutide and tirzepatide, reduce the severity of OSA, reflected in decreases in the AHI, in patients with obesity and/or T2DM. Excessive adiposity is one of the main reversible etiological factors of the disease, and the reduction induced by these drugs could decrease compression of the upper airway and prevent its collapse during sleep. Furthermore, glycemic control and sustained weight loss are factors that indirectly improve sleep architecture. For this reason, the benefit observed in this population may be more related to weight loss and the metabolic improvement associated with treatment.

To date, the evaluated population has been relatively homogeneous: patients with moderate-to-severe OSA associated with obesity and/or metabolic alterations. Consequently, it is still unclear whether these medications would be equally effective in patients with OSA without metabolic syndrome, with normal body mass index, or with overweight only. Likewise, patients with mild OSA have not been studied. This limits the generalization of findings to less severe forms of the disease and without metabolic syndrome as a comorbidity. These findings are clinically relevant because many patients present difficulties adhering to CPAP or sustained weight loss interventions. In this context, GLP-1 agonists could represent a complementary or alternative therapeutic strategy in patients with obesity and OSA who do not tolerate CPAP. Finally, studies with prolonged follow-up are required in order to determine the necessary duration of GLP-1 receptor agonist therapy that allows patients to maintain a stable weight and persistent improvement in apnea/hypopnea indices. Thus, based on the current available evidence, the long-term durability of these effects remains uncertain.

Current clinical evidence therefore suggests that liraglutide and tirzepatide are medications that can help improve severe/moderate OSA in patients with obesity or metabolic syndrome, and at present it cannot be extrapolated to either another type of apnea or another population. Their high economic cost and the incidence of gastrointestinal adverse reactions that require cautious dose escalation to ensure treatment adherence can be a limiting factor for the use of this therapy.

## 7. Future Directions and Unanswered Questions

Despite the growing clinical evidence supporting GLP-1-based therapies in obesity-associated OSA, several fundamental questions remain unanswered. Addressing these gaps will be essential to define their mechanisms, target populations, and position relative to airway-directed treatment.

According to the available clinical evidence [30,118,119,120,122], it has not been definitively established whether improvements in OSA are entirely mediated by weight loss. Although weight loss is currently considered the principal mediator, no clinical study has demonstrated a direct pharmacological respiratory effect that is clearly independent of it. No formal mediation analyses have been performed; studies have not established whether AHI improvement precedes weight reduction; and proposed mechanisms involving ventilatory stability, inflammation, autonomic function, or leptin signaling remain largely hypothetical. Generalizability is also uncertain. The major efficacy data relate to adults with obesity and/or T2DM and moderate-to-severe OSA. Dedicated trials in non-obese populations and studies incorporating physiological endotypes are required to determine whether any clinically meaningful weight-independent effect exists.

A further unresolved question concerns comparative efficacy across drug classes. Both selective GLP-1RAs and dual GIP/GLP-1 receptor agonism improve OSA severity, but no head-to-head trial has established superiority. In SURMOUNT-OSA, tirzepatide produced mean AHI reductions of 25.3 events/h in participants not receiving PAP and 29.3 events/h in those continuing PAP [30]. In SCALE Sleep Apnea, liraglutide produced a mean reduction of 12.2 events/h compared with 6.1 events/h with placebo [118]. These cross-trial differences may reflect pharmacological potency, degree of weight loss, treatment duration, population characteristics, or study design and should not be interpreted as a direct comparative estimate.

An equally important question is how these therapies should be integrated with CPAP. Current evidence supports GLP-1-based pharmacotherapy as an obesity-directed strategy rather than a universal replacement for airway stabilization [142]. SURMOUNT-OSA included one trial in participants not using CPAP and another in participants continuing PAP, with substantial AHI reductions in both settings [30]. By contrast, the O’Donnell study showed substantially greater respiratory control with CPAP alone or combined with liraglutide than with liraglutide alone [119]. Tirzepatide may therefore be used without CPAP in eligible adults within its approved indication or as an adjunct to CPAP, while treatment selection should consider OSA severity, symptoms, hypoxic burden, anatomy, cardiometabolic risk, and CPAP tolerance [143]. No validated physiological biomarkers or endotypes currently predict response to GLP-1-based therapy. Imaging of upper-airway and lingual fat and direct assessment of P_crit_, loop gain, arousal threshold, and muscle compensation remain important research priorities [144].

Taken together, these gaps point to a common need to conduct mechanistic human trials that integrate physiological, anatomical, and pharmacological outcomes. Future studies should incorporate imaging modalities such as MRI or CT of the upper airway alongside direct measurement of endotypes, such as P_crit_, loop gain, and arousal threshold, in relation to polysomnographic outcomes, ideally supported by formal mediation analyses to determine the extent to which treatment response is independent of weight loss.

## 8. Conclusions

OSA is a multifactorial disorder in which anatomical, neuromuscular, metabolic, and neuroendocrine disturbances interact. GLP-1-based therapies have emerged as an important obesity-directed strategy for moderate-to-severe OSA, with clinical benefits most consistently explained by weight loss, reduced mechanical loading, and associated cardiometabolic improvement. Tirzepatide has the strongest evidence, including two phase 3 trials and an FDA indication for adults with obesity and moderate-to-severe OSA [30,145]; selective GLP-1RAs have more limited and off-label evidence for this condition.

Preclinical evidence supports biological plausibility but relies mainly on intermittent hypoxia, obesity, metabolic disease, and cellular models that do not reproduce the complete OSA syndrome. Clinical evidence consistently shows reductions in body weight and AHI, yet formal mediation analyses and direct studies of OSA endotypes are lacking. Anti-inflammatory biomarker improvement has been observed, but oxidative stress, leptin, autonomic, central respiratory, and direct upper-airway neuromuscular mechanisms remain unconfirmed in patients with OSA.

Clinical implementation should remain within approved indications and under appropriate medical supervision, with attention to gastrointestinal adverse effects, lean-mass loss, access, treatment discontinuation, weight regain, and possible recurrence of OSA [145,146,147,148]. Future trials should integrate polysomnography, upper-airway imaging, physiological endotyping, and formal mediation analysis to determine the relative contributions of weight loss and additional mechanisms and to define long-term durability and optimal combination with CPAP. Overall, although several biologically plausible mechanisms have been proposed, current clinical evidence does not yet demonstrate direct modulation of upper-airway physiology or ventilatory control independent of weight loss, underscoring an important direction for future mechanistic research.

## Figures and Tables

**Table 1:** Major physiological endotypes of obstructive sleep apnea.

Physiological Endotype	Definition	Pathophysiological Relevance	Contribution to OSA Severity	Potential Mechanism Related to GLP-1RA
Upper-airway collapsibility [28]	Passive tendency of the pharynx to narrow or close during sleep, quantified by P_crit_, V_passive_	Reflects anatomical restriction, regional adiposity and reduced tracheal traction	↑ P_crit_: increases the likelihood and persistence of obstruction	Weight control →reductions in cervical, lingual, peripharyngeal and visceral adiposity
Loop gain [29]	Magnitude of the ventilatory response to a preceding respiratory disturbance	Determines stability of respiratory feedback control	↑ Loop gain: ↑ ventilatory overshoot, ↑ recurrent events, ↓ respiratory drive, hypocapnia	Possible modulation through metabolic, autonomic, leptin, or chemoreflex pathways
Respiratory arousal threshold [30]	Intensity of respiratory effort required to trigger cortical arousal	Determines when an obstructive event is interrupted	↓ Respiratory arousal threshold: premature arousal and instability ↑ Respiratory arousal threshold: prolonged obstruction	Remains to be established
Upper-airway muscle responsiveness [31]	Ability of pharyngeal dilator muscles to respond effectively to airway narrowing and negative pressure, measured by v_ive_−V_passive_	Contributes to neuromuscular compensation for anatomical vulnerability	Delayed or insufficient recruitment favors persistent collapse	Possible indirect improvement through weight loss, reduced inflammation, and altered leptin signaling; no direct evidence

P_crit:_ critical pressure at which the airway collapses. V_passive:_ ventilation level when the airway is completely relaxed. V_active:_ ventilation achieved with active upper-airway muscle recruitment. ↑: increase, ↓: decrease.

**Table 2:** Level and OSA-specificity of evidence supporting proposed mechanisms of GLP-1-based therapies in obstructive sleep apnea.

Proposed Mechanism	Evidence Described in the Manuscript	Level and OSA-Specificity of Evidence	Interpretation	References
Weight loss and reduced mechanical loading	Randomized trials in obesity-associated OSA demonstrate reductions in body weight and AHI with liraglutide and tirzepatide.	Direct human clinical evidence for weight loss and AHI reduction; mediation is inferred rather than formally established.	Best-supported and probably dominant mechanism, but a formal weight-loss mediation analysis is lacking.	[30,118,119,120,121]
Reduction in regional and upper-airway adiposity	Regional adiposity is associated with OSA severity. GLP-1-based therapies reduce visceral and ectopic fat; reduced tongue fat has been reported outside OSA, and endotypic OSA evidence is limited to a case report.	Preclinical and indirect human evidence, mostly outside OSA; OSA-specific physiological evidence limited to one patient.	Plausible extension of weight loss, but no trial has shown that reduction in tongue or peripharyngeal fat mediates AHI improvement.	[24,32,90,91,92,93,94,95,122]
Reduction in systemic inflammation	Cellular and animal studies show anti-inflammatory signaling. Human OSA trials measured hsCRP, with significant reduction in SURMOUNT-OSA; an intermittent-hypoxia murine study showed reduced IL-6 and IL-1β transcription.	Direct OSA biomarker evidence plus cellular, animal, and non-OSA human evidence; no proof of respiratory mediation.	Inflammatory biomarker improvement is demonstrated, but an independent causal contribution to AHI reduction is unconfirmed.	[30,110,111,112,113,114,115,116,117,118,123,124,125,126,127]
Reduction in oxidative stress	Oxidative stress is central to intermittent-hypoxia pathophysiology, and GLP-1 receptor activation reduces ROS-related signaling in cellular and metabolic models.	OSA pathophysiological evidence plus cellular/preclinical treatment evidence; no direct mechanistic treatment evidence in patients with OSA	Biologically plausible and hypothesis-generating.	[40,41,44,114,115,116]
Modification of leptin signaling	Leptin influences respiratory drive and upper-airway neuromuscular control. GLP-1–leptin interactions and altered leptin expression have been described mainly in experimental models.	Predominantly preclinical and indirect human evidence; no direct evidence that this pathway mediates clinical OSA improvement.	Plausible but unconfirmed therapeutic mechanism.	[33,34,53,54,55,56,57,58,59,60,61,87,124]
Autonomic nervous system modulation	GLP-1 receptor activation influences vagal and autonomic pathways, while sympathetic overactivation contributes to OSA-related metabolic and cardiovascular dysfunction.	General mechanistic and non-OSA evidence; no direct autonomic mediation study in treated patients with OSA.	Plausible but not established as a mediator of AHI reduction.	[40,41,47,50,77,78,79,80,81]
Central respiratory and chemoreflex effects	Vagal GLP-1 signaling projects to brainstem and hypothalamic regions, providing a neuroanatomical rationale for possible respiratory effects.	Neuroanatomical and mechanistic evidence; no confirmatory human mechanistic evidence in OSA.	Hypothetical in human OSA.	[81]
Direct upper-airway neuromuscular effect	GLP-1 receptor expression has not been demonstrated in human genioglossus or pharyngeal skeletal muscle. A single-patient report described improved muscle compensation during tirzepatide treatment.	No direct receptor expression or pharmacological evidence; OSA-specific physiological evidence limited to a case report.	A direct muscular action is speculative; any effect is more likely indirect.	[32,74,75]

OSA: obstructive sleep apnea; GLP-1: glucagon-like peptide-1; AHI: apnea–hypopnea index; hsCRP: high-sensitivity C-reactive protein; IL-6: interleukin-6; IL-1β: interleukin-1 beta; ROS: reactive oxygen species.

**Table 3:** Clinical outcomes of GLP-1-based therapies in patients with obstructive sleep apnea.

Authors and Publication Date	Study Type	Total Sample	Population Included	Population Excluded	Age	Males %	Baseline AIH	Intervention and Duration of Treatment	Primary Outcome
Malhotra et al. 2024 [30]	RCT	234	Adults with moderate-to-severe OSA and obesity	Diabetes, change in body weight > 5 kg in the 3 months before screening, planned surgery for OSA or obesity, a diagnosis of central or mixed sleep apnea, and major craniofacial abnormality	Trial 1: 47.9 years	67.1%	51.5 events per hour.	Trial 1: 52 weeks of treatment with tirzepatide starting at 2.5 mg weekly and escalating every 4 weeks to reach a maximum tolerated dose of 10–15 mg	Treatment-regimen estimand: Tirzepatide arm: −25.3 events/hour (95% CI, −29.3 to −21.2) Placebo arm: −5.3 events/hour (95% CI, −9.4 to −1.1); estimated treatment difference: −20.0 events/hour (95% CI, −25.8 to −14.2; *p* < 0.001).
Malhotra et al. 2024 [30]	RCT	235	Adults with moderate-to-severe OSA and obesity	Diabetes, change in body weight > 5 kg in the 3 months before screening, planned surgery for OSA or obesity, a diagnosis of central or mixed sleep apnea, and major craniofacial abnormality	Trial 2: 51.7 years.	72.3%	49.5 events per hour.	Trial 2: 52 weeks of treatment with CPAP therapy + Tirzepatide starting at 2.5 mg weekly and escalating every 4 weeks to reach a maximum tolerated dose of 10–15 mg	Treatment-regimen estimand: Tirzepatide arm: −29.3 events/hour (95% CI, −33.2 to −25.4) Placebo arm: −5.5 events/hour (95% CI, −9.9 to −1.2). Estimated treatment difference: −23.8 events/hour (95% CI, −29.6 to −17.9; *p* < 0.001).
Blackman et al. 2016 [118]	RCT	276	Adults aged 18–64 years with BMI ≥ 30 kg/m^2^, stable weight, moderate-to-severe OSA, and unable/unwilling to use CPAP.	Patients with central sleep apnea or diabetes were excluded.	Liraglutide: 48.6 ± 9.9 Placebo: 48.4 ± 9.5	Liraglutide:(71.7) % Placebo (72.1)%	Mean baseline AHI of approximately 49 events	32 weeks of treatment with liraglutide starting at 0.6 mg daily and escalating 0.6 mg every week to reach a maintenance dose of 3.0 mg daily.	At week 32, AHI reduction was greater with liraglutide vs. placebo (−12.2 ± 1.8 vs. −6.1 ± 2.0 events/hour); estimated treatment difference: −6.1 events/hour (95% CI, −11.0 to −1.2; *p* = 0.015).
O’Donnell et al., 2024 [119]	RCT	30	Adults without diabetes, with moderate-to-severe OSA and BMI between 30 and 40 kg/m^2^	Patients with diabetes and BMI over 40 kg/m^2^	Total: 50 ± 7 Liraglutide: 50 ± 10 CPAP: 49 ± 8 Combination: 51 ± 4	Total: 80% Liraglutide: 80% CPAP: 91% Combination: 67%	Total: 50 ± 19 Liraglutide: 54 ± 21 CPAP: 48 ± 20 Combination: 43 ± 17	24 weeks of treatment with liraglutide starting at 0.6 mg daily and escalating 0.6 mg every week until a daily dose of 3 mg was reached. A group received combination therapy with CPAP	At week 24, AHI reduction was greater than with CPAP alone (3 ± 3 events/hour) and combination therapy (5 ± 5 events/hour) compared to liraglutide alone (42 ± 16 events/hour).
Jiang et al., 2023 [120]	RCT	90	Adults 18–75 years with T2DM, moderate to severe OSA (AHI > 15), current CPAP treatment, and no current DPP-IV inhibitor or GLP-1RA use	Pregnancy/breastfeeding, contraindications to DPP-IV inhibitors or GLP-1RAs, NYHA III–IV heart failure, CKD stage 4–5, pancreatitis history, infections, thyroid disease, malignancy, unwillingness to follow-up	Liraglutide: 55.7 ± 7.4 years Control: 54.8 ± 5.5 years	Liraglutide: 34/44 men (77.3%) Control: 29/45 men (64.4%)	Liraglutide: 31.0 ± 7.3 events/h Control: 30.1 ± 6.22 events/h	12-week treatment with liraglutide starting at 0.6 md daily and escalating 0.6 mg weekly to reach a daily dose of 1.2–1.8 mg.	AHI decreased from 31.0 ± 7.3 to 26.1 ± 7.1 events/h in liraglutide group (*p* < 0.001) vs. no significant change in controls (30.1 ± 6.22 to 31.6 ± 6.9; *p* = 0.246).

RCT: double-blind randomized controlled trial; AHI: apnea–hypopnea index; OSA: obstructive sleep apnea; BMI: body mass index; CPAP: continuous positive airway pressure; CI: confidence interval; CKD: chronic kidney disease; GLP-1RA: glucagon-Like peptide-1 receptor agonist; NYHA: New York Heart Association; T2DM: type 2 diabetes mellitus.

## Data Availability

No new data were created or analyzed in this study. Data sharing is not applicable to this article.
